# DDB2 regulates DNA replication through PCNA-independent degradation of CDT2

**DOI:** 10.1186/s13578-021-00540-5

**Published:** 2021-02-08

**Authors:** Xiaojun Wu, Min Yu, Zhuxia Zhang, Feng Leng, Yue Ma, Ni Xie, Fei Lu

**Affiliations:** 1 State Key Laboratory of Chemical Oncogenomics, Key Laboratory of Chemical Genomics, Peking University Shenzhen Graduate School, 518055 Shenzhen, China; 2 Research Center for Protein and Cell-based Drugs, Institute of Biomedicine and Biotechnology, Shenzhen Institutes of Advanced Technology, Chinese Academy of Sciences, 518055 Shenzhen, China; 3 Biobank, Shenzhen Second People’s Hospital, The First Affiliated Hospital of Shenzhen University, Health Science Center, 518035 Shenzhen, China

**Keywords:** DDB2, CDT2, Protein degradation, DNA replication, Ubiquitin ligase, Cancer

## Abstract

**Background:**

Targeting ubiquitin-dependent proteolysis is one of the strategies in cancer therapy. CRL^CDT2^ and CRL^DDB2^ are two key E3 ubiquitin ligases involved in DNA replication and DNA damage repair. But CDT2 and DDB2 are opposite prognostic factors in kinds of cancers, and the underlining mechanism needs to be elucidated.

**Methods:**

Small interfering RNAs were used to determine the function of target genes. Co-immunoprecipitation (Co-IP) was performed to detect the interaction between DDB2 and CDT2. Immunofluorescence assays and fluorescence activating cell sorting (FACS) were used to measure the change of DNA content. In vivo ubiquitination assay was carried out to clarify the ubiquitination of CDT2 mediated by DDB2. Cell synchronization was performed to arrest cells at G1/S and S phase. The mechanism involved in DDB2-mediated CDT2 degradation was investigated by constructing plasmids with mutant variants and measured by Western blot. Immunohistochemistry was performed to determine the relationship between DDB2 and CDT2. Paired two-side Student’s t-test was used to measure the significance of the difference between control group and experimental group.

**Results:**

Knockdown of DDB2 stabilized CDT2, while over-expression of DDB2 enhanced ubiquitination of CDT2, and subsequentially degradation of CDT2. Although both DDB2 and CDT2 contain PIP (PCNA-interacting protein) box, PIP box is dispensable for DDB2-mediated CDT2 degradation. Knockdown of PCNA had negligible effects on the stability of CDT2, but promoted accumulation of CDT1, p21 and SET8. Silencing of DDB2 arrested cell cycle in G1 phase, destabilized CDT1 and reduced the chromatin loading of MCMs, thereby blocked the formation of polyploidy induced by ablation of CDT2. In breast cancer and ovarian teratoma tissues, high level of DDB2 was along with lower level of CDT2.

**Conclusions:**

We found that CRL4^DDB2^ is the novel E3 ubiquitin ligases of CDT2, and DDB2 regulates DNA replication through indirectly regulates CDT1 protein stability by degrading CDT2 and promotes the assembly of pre-replication complex. Our results broaden the horizon for understanding the opposite function of CDT2 and DDB2 in tumorigenesis, and may provide clues for drug discovery in cancer therapy.

## Background

Genome instability and gene amplification are two features of cancer cells. To ensure the high fidelity of DNA replication and the stability of genome, DNA replication occurs once and only once in one cell cycle. Eukaryotic cells have developed a series of mechanisms to prevent the abnormal replication of DNA and maintain the stability of genome [1, 2]. One of the key regulations of DNA replication is the degradation of CDT1 once DNA replication is initiated. The proteolysis of CDT1 is mediated by CUL1^Skp2^ ubiquitin ligase and CRL4^CDT2^ ubiquitin ligase during S phase and under UV radiation [3, 4].

CRL4^CDT2^ ubiquitin ligase regulates cell proliferation by degrading important substrates such as CDT1, p21 and SET8, which involves in DNA replication licensing, cell cycle regulation, and chromatin modification [5–7]. As a substrate receptor for CRL4 ubiquitin E3 ligase, CDT2 interacts with PCNA and promotes the proteolysis of most substrates in PCNA-dependent manner [6–8]. CDT2 is highly expressed in lung cancer, breast cancer, colon cancer and Ewing sarcoma, and relates to the poor survival of cancer patients [9–12]. Silencing of CDT2 induces the apoptotic death of human cancer cells from different tissues, but not non-transformed human cells and primary cells, which may due to the replicative stress and DNA damage [13]. Targeting to proteolysis is one of the strategies in cancer treatment. MLN4924 is the specific inhibitor of the NEDD8 activating enzyme (NAE), can inhibits growth of cancer cells and suppresses tumor growth in nude mice [14]. MLN4924 can inhibit the activity of CRL4^CDT2^ ubiquitin ligase to stabilize CDT1 and trigger checkpoint activation, apoptosis, and senescence in cancer cells [15]. In ovarian cancer cells, knockdown of CDT2 but not DCAF1 phenocopies the effects of MLN4924, and silencing of CDT1 partially rescues apoptotic death induced by MLN4924 [16], while the pharmacological effect of MLN4924 is independent of p21 or SET8 [14], indicating special role of CDT2 and CDT1 in cancer development.

The protein level of CDT2 is increased during G1/S phase, and decreased in mitosis via the APC/C-Cdh1 mediated degradation [11]. It is reported that CDT2 is polyubiquitylated by the CRL4A and CRL1 E3 ubiquitin ligase, and degraded by CRL1^FBXO11^ ubiquitin ligase [17]. CRL1^FBXO11^ mediated CDT2 degradation can stabilize p21 and SET8 but not CDT1 [17]. In this study, we reported that CRL4^DDB2^ is a new E3 ubiquitin ligase targeting CDT2 for its proteolysis. DNA damage binding protein 2 (DDB2), another substrate receptor for CRL4 E3 ubiquitin ligase [18–22], is originally identified as damage-specific DNA binding protein, and involves in the early step of DNA damage recognition of nucleotide excision repair (NER), which is induced by UV radiation [23–27]. Recently, increasing evidences suggest that DDB2 plays an important role in suppressing tumorigenesis [19, 28–32]. DDB2 is low expressed in skin cancer, breast cancer, colon cancer, prostate cancer, and ovarian cancer [18, 29, 30, 33–35]. DDB2-deficient mice have the high rate to develop malignant tumors compared to their XP-deficient littermates [36]. Here we evidenced that silencing of DDB2 blocks the occurrence of DNA re-replication induced by CDT2 knockdown, and DDB2 can indirectly regulate CDT1 protein level through degrading CDT2 in PCNA-independent manner. Thus, our study provided new angle on understanding the opposite role of CDT2 and DDB2 in tumorigenesis.

## Results

### The protein stability of CDT2 is regulated by DDB2

**Fig. 1:**
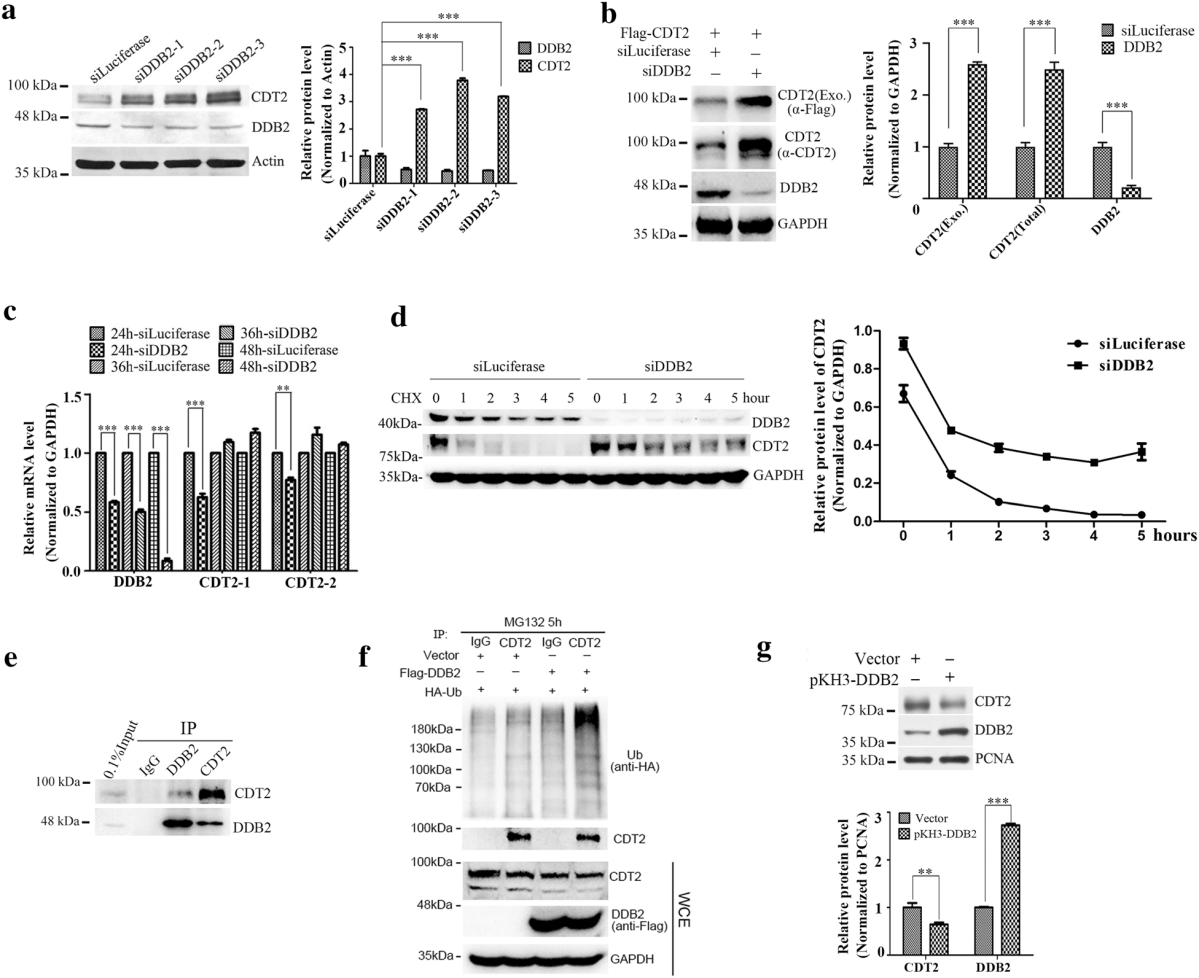
CRL^DDB2^ is a new E3 ubiquitin ligase of CDT2. **a** The protein level of CDT2 was accumulated when DDB2 was silenced. HCT116 cells were transfected with luciferase and DDB2 specific siRNAs for 48 h and subjected to Western blot. Actin was taken as loading control. Right panel: the relative protein levels of CDT2 and DDB2 were quantified by Gel-pro analyzer 4.0, and the P value was calculated by the two-side Student’s t-test (*** indicated P < 0.001). The error bars denoted standard deviation (SD). **b** Silencing of DDB2 accumulated exogenous CDT2. HCT116 cells were treated with indicated siRNAs for 18 h, and then transfected with pCMV10-3Flag-CDT2 by polyetherimide and cultured for another 40 h. The protein levels of CDT2 and DDB2 were analyzed by Western blot. Exogenous CDT2 was detected by anti-Flag antibody. The relative protein level of CDT2 was measured using Gel-pro analyzer 4.0 and plotted on the right panel. *** indicated P < 0.001. The error bar indicated SD. **c** DDB2 had negligible effects on CDT2 transcription. HCT116 cells were transfected with indicated siRNAs, and mRNAs were extracted at 24 h, 36 h and 48 h respectively. The mRNA levels of interested genes were measured by RT-qPCR. Two pairs of specific primers targeting to CDT2 were used to quantify mRNA level of *CDT2*. Student’s t-test was used to calculate P value (** indicated P < 0.01, *** indicated P < 0.001). The error bars denoted SD. **d** Down regulation of DDB2 prolonged the half-life of CDT2. HCT116 cells were transfected with specific siRNAs for 48 h, and then treated with 100 µg/mL CHX for indicated times. The protein levels of CDT2 and DDB2 were measured by specific antibodies. The relative protein level of CDT2 was plotted on the right panel. The error bars denoted SD. **e** DDB2 and CDT2 interacted mutually. HCT116 cells were harvested with lysis buffer after treated with Mg132 (10 µg/mL) for 4 h, and then immunoprecipitated with NRS (normal rabbit serum), anti-CDT2 and anti-DDB2 antibodies respectively. The protein levels of interest were detected by Western blot. **f** CRL4^DDB2^ complex promoted CDT2 polyubiquitination in vivo. HCT116 cells were co-transfected with pKH3-Ub and pCMV10-3Flag-DDB2 or pCMV10-3Flag (Vector) for 48 h, and lysed after MG132 treatment for 5 h. Cell lysate was immunoprecipitated with NRS or anti-CDT2 antibody, and immunocomplexes were analyzed with indicated antibodies. Immunoblots of whole cell extracts were shown at the bottom. **g** DDB2 promoted CDT2 degradation. HCT116 cells were transfected with pKH3 (Vector) or pKH3-DDB2 for 48 h, and the protein levels of interest were detected by Western blot. Bottom panel, the relative protein levels of CDT2 and DDB2 was quantified. The error bars represent SD

Both CDT2 and DDB2 are DCAFs, and act as the substrate receptor for CRL4 E3 ubiquitin ligase. During investigating the substrates of DDB2, we found that CDT2 protein level was significantly accumulated after knocking down DDB2 (Fig. 1a). To avoid off-target effects, we designed three pairs of siRNAs targeting to DDB2 and the similar results were obtained (Fig. 1a). Then we asked that the expression of CDT2 regulated by DDB2 was at transcriptional level or at post-translational level. It is reported that DDB2 transcriptional regulates *IκBα* and *SOD2* in breast cancer cells [34, 35], and *Snail*, *Zeb1* and *VEGF* in colon cancer [30]. To clarify whether DDB2 is also a transcriptional regulator of CDT2, we constructed plasmid expressing 3Flag tagged CDT2, and examined the mRNA level of CDT2 after DDB2 knocking down. As shown in Fig. 1b, the protein level of exogenous CDT2, which did not contain the promotor region of CDT2, was significant increased when DDB2 was silenced (Fig. 1b). The change of exogenous CDT2 protein level was consistent with that of endogenous CDT2 (Fig. 1b). Meanwhile, real-time quantitative PCR analysis revealed that the mRNA level of *CDT2* was decreased but not increased at 24 h post transfection with DDB2 siRNAs, and then slightly increased at 36 h or 48 h after silencing of DDB2 (Fig. 1c). Furthermore, we monitored the half-life of CDT2 using CHX to inhibit the de novo protein synthesis. The protein level of CDT2 was decreased rapidly in luciferase siRNA treated cells with a half-life around half an hour, and silencing of DDB2 significantly prolonged the half-life of CDT2 (Fig. 1d). Taken together, our data suggested that DDB2 regulates the expression of CDT2 at post-translational level but not transcriptional level.

### DDB2 interacts with CDT2 and mediates its ubiquitination

It is well known that DDB2 is a substrate receptor for CRL4 E3 ubiquitin ligase, and promotes proteolysis of AR, H2A, XPC, p27 and itself [20, 22, 37–40]. We tested whether CDT2 is directly ubiquitinated by CRL4^DDB2^ complex. Firstly, we checked the interaction between CDT2 and DDB2 by co-immunoprecipitation. The result showed that DDB2 associated with CDT2 and vice versa in HCT116 cells (Fig. 1e). It indicated that CDT2 forms a complex with DDB2. Then in vivo ubiquitination assay was performed to determine whether DDB2 can ubiquitinate CDT2 or not. The plasmids expressing HA-tagged ubiquitin were co-transfected with pCMV10-3Flag-DDB2 or pCMV10-3Flag (Vector) into HCT116 cells for 48 h. And then cells were treated with MG132 for 5 h and lysed for immunoprecipitation using anti-CDT2 antibody (rabbit IgG was taken as control). As shown in Fig. 1f, over-expression of DDB2 significantly increased the ubiquitinated CDT2 (Fig. 1f). It was consistent with the result that over-expression of DDB2 promotes degradation of CDT2 (Fig. 1g). Thus, CRL4 ^DDB2^ is a newly identified E3 ubiquitin ligase for CDT2.

### The ubiquitination-mediated degradation of CDT2 by CRL4 ^DDB2^ is independent of PCNA

**Fig. 2:**
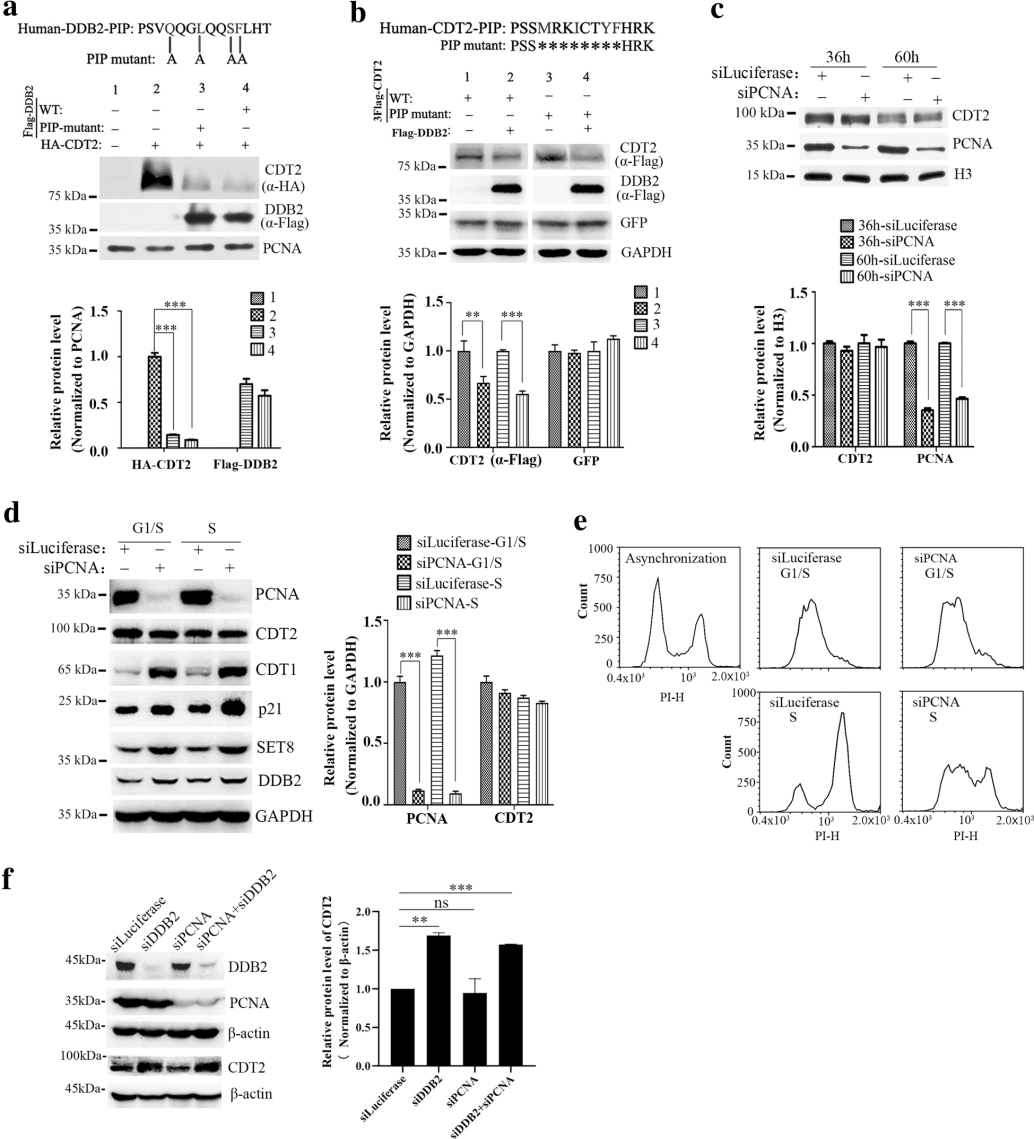
PCNA is dispensable for degradation of CDT2 mediated by DDB2. **a** Both wild-type DDB2 and PIP-mutant DDB2 promoted CDT2 degradation. Upper panel: the mutated sites in DDB2 PIP box. Middle panel: HCT116 cells were co-transfected with pKH3-CDT2 and pCMV10-3Flag-DDB2 or pCMV10-3Flag-DDB2-PIP-mutant plasmids for 40 h, and cell lysates were blotted by indicated antibodies. Lower panel: the relative protein levels of interest were measured by Gel-pro analyzer 4.0. The significance between experiment group (co-transfection of CDT2 and DDB2 or DDB mutant) and control group (only CDT2 transfection) was calculated by two-side Student’s t-test. *** Denoted P < 0.001. **b** DDB2 promoted degradation of both wild-type CDT2 or PIP mutant CDT2. Upper panel: the sequence of CDT2 PIP box and the amino acids deleted. Middle panel: HCT116 cells were co-transfected with pCMV10-3Flag-DDB2 and pCMV10-3Flag-CDT2 or pCMV10-3Flag-CDT2-PIP-mutant plasmids for 48 h, and cells were harvested for Western blot. GFP was co-transfected to indicate transfection efficiency. Proteins of interest were blotted by specific antibodies. Lower panel: the relative protein level of exogenous CDT2 was measured by Gel-pro analyzer 4.0 (lower panel). The P value was evaluated by the two-side Student’s t-test. *** Denoted P < 0.001. **c** Silencing of PCNA could not accumulate CDT2 protein. HCT116 cells were transfected with siRNAs specific targeting to PCNA, and harvested for Western blot analysis. The relative protein levels were normalized and plotted on the lower panel. *** Denoted P < 0.001. **d** Knockdown of PCNA could not up-regulate CDT2 at G1/S or S phase, but accumulated CDT1, p21 and SET8. HCT116 cells were transfected with luciferase and PCNA siRNAs, and synchronized at G1/S and S phase by double thymidine treatment. Proteins of interest were detected by indicated antibodies, and relative protein levels were measured by Gel-pro analyzer 4.0 (right panel). The P value was evaluated by the two-side Student’s t-test (** denoted P < 0.01; *** denoted P < 0.001). **e** Cell synchronization was confirmed by FACS. **f** PCNA has no effect on DDB2-mediated CDT2 degradation. HCT116 cells were transfected with luciferase, DDB2, PCNA and DDB2 + PCNA siRNAs for 60 h and total cell lysate was analyzed using indicated antibodies. The relative protein level of CDT2 was measured and plotted on the right panel. The P value was evaluated by the two-side Student’s t-test (** denoted P < 0.01; *** denoted P < 0.001). The error bars indicated SD

CRL4^DDB2^ mediated degradation of DDB2 and p21 is PCNA-dependent, and PIP box contributes to the interaction between DDB2 and PCNA [41]. In addition, CDT2 is also a PIP box containing protein, and can directly bind to PCNA through the PIP box [6, 42]. Our previous work showed that PIP box in CDT2 is essential for CDT1 degradation [42]. We supposed that the ubiquitination-mediated degradation of CDT2 by CRL4^DDB2^ ligase is in a PCNA-dependent manner. To test our hypothesis, we constructed the plasmids expressing wild type or PIP box mutated DDB2 or CDT2 (Fig. 2a upper panel and 2b upper panel). To our surprise, the data showed that over-expression of both wild type DDB2 and PIP box mutated DDB2 significantly decreased protein level of CDT2 (Fig. 2a), and over-expression of DDB2 had the similar effects on degrading wild type CDT2 and PIP box mutated CDT2 (Fig. 2b). Thus, our data suggested that PIP box is dispensable for the degradation of CDT2 mediated by CRL4^DDB2^ ubiquitin ligase.

To further validate the results, we designed two pairs of siRNAs specifically targeting to PCNA, and examined the protein level of CDT2. Interestingly, CDT2 did not accumulate but slightly decreased in PCNA deficient cells (Fig. 2c). According to previous report, knockdown of PCNA induces S/G2 phase arrest, which is the window for CRL1^FBXO11^ mediated degradation of CDT2 [17]. To eliminate the possible influence induced by cell cycle arrest, we synchronized cells at G1/S phase and S phase. Western blot analysis showed that the protein level of CDT2 was not increased but slightly decreased after PCNA silencing in both G1/S phase and S phases (Fig. 2d), which may due to a little increase of DDB2 after PCNA knocking down (Fig. 2d). The efficiency of cell synchronization was confirmed by FACS (Fig. 2e). But the protein levels of CDT1, p21 and SET8 were remarkably increased after PCNA knockdown (Fig. 2d), which is consistent with previous reports that PCNA is required for proteolysis of CDT1, p21 and SET8. Moreover, knockdown of PCNA did not affect CDT2 accumulation induced by DDB2 silencing (Fig. 2f). Taken together, our studies suggested that PCNA is dispensable for the degradation of CDT2 mediate by CRL4^DDB2^ ubiquitin ligase.

### Silence of DDB2 blocks DNA re-replication induced by abolishment of CDT2

**Fig. 3:**
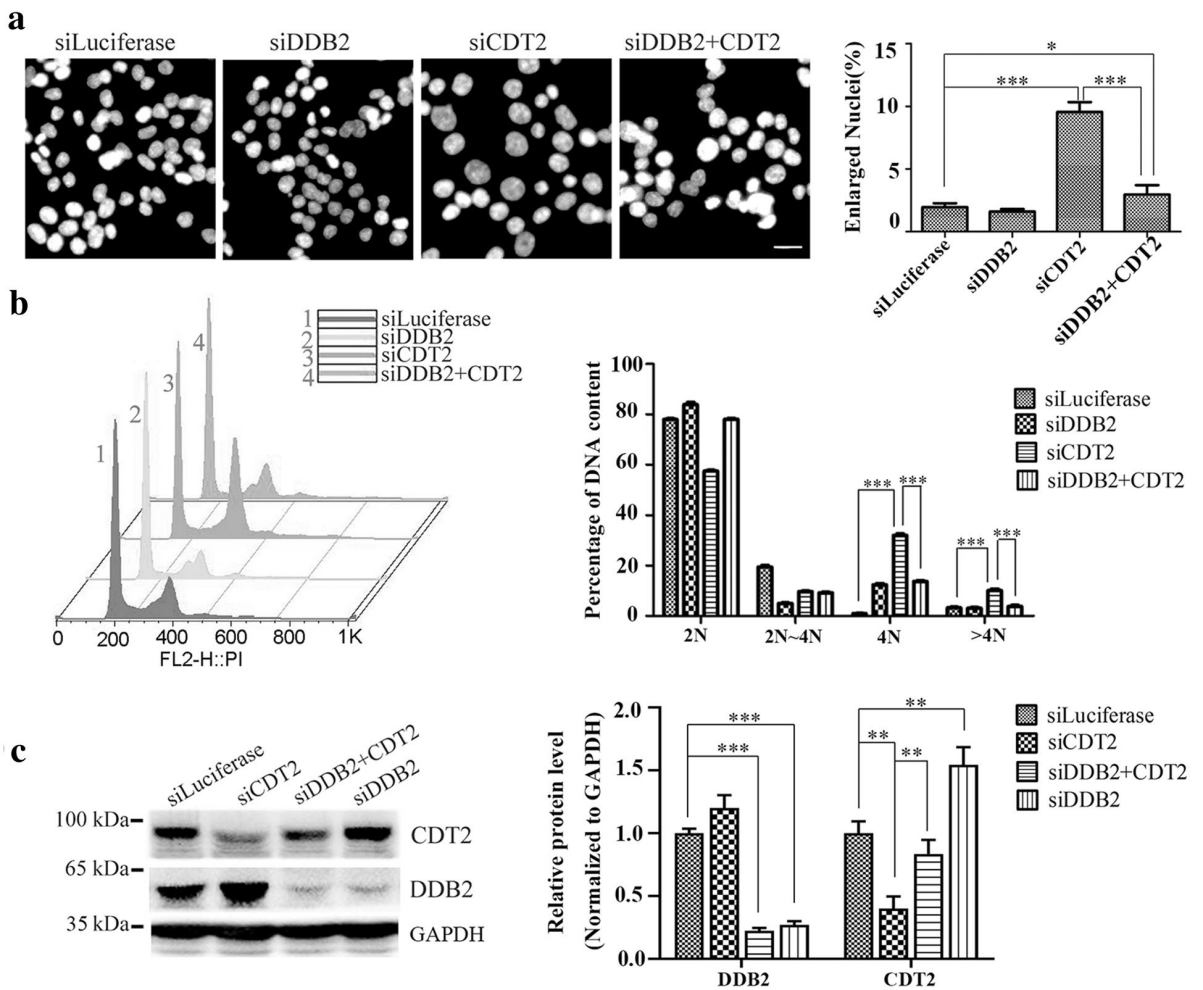
Reducing the expression of DDB2 suppresses re-replication induced by CDT2 deficiency. **a** Silencing of DDB2 inhibited the formation of giant nuclei induced by CDT2 knockdown. HCT116 cells were transfected with indicated siRNAs for 48 h and fixed with 4% paraformaldehyde. DNA was stained by DAPI (4′,6-diamidino-2-phenylindole). Scale bar: 50 µm. Right panel: the percentages of enlarged nuclei in** a** the error bars indicated SD. Significant difference was observed between CDT2 siRNA- and luciferase siRNA- or DDB2 + CDT2 double siRNA-treated cells, which was evaluated by the two-side Student's t-test (*** indicated P < 0.001). **b** FACS analysis of DNA contents in **a**. The percentages of DNA contents were presented in the right panel. The significant difference was observed between CDT2 siRNA- and luciferase siRNA- or DDB2 + CDT2 double siRNA-treated cells with 4N and > 4N DNA contents, which was evaluated by the two-side Student's t-test (*** indicated P < 0.001). **c** The efficacy of siRNAs in **a** and **b** were analyzed by Western blot. Right panel: The relative protein levels were measured using Gel-pro analyzer 4.0, and the P values were evaluated using the two-side Student's t-test (* denoted P < 0.05, ** denoted P < 0.01, *** denoted P < 0.001). The error bars indicated SD

Since CRL4^CDT2^ ubiquitin ligase degrades CDT1 once DNA replication initiated to ensure DNA replicated only once in one cell cycle, and depletion of CDT2 can induce DNA re-replication due to CDT1 stabilization. We asked whether DDB2 can suppress DNA re-replication or not, which is induced by CDT2 knockdown. As expected, down-regulation of CDT2 caused the occurrence of DNA re-replication and the formation of giant nuclei (Fig. 3a, b). Co-silencing of DDB2 blocked both DNA re-replication and giant nuclei formation induced by knocking down CDT2 (Fig. 3a, b). As shown in FACS analysis, silencing of CDT2 increased the number of cells with 4N DNA content and the formation of polyploidy (> 4N), but when down-regulation of DDB2 at the same time, the DNA contents were rescued (Fig. 3b). The efficacy of siRNAs was confirmed by Western blot using specific antibodies (Fig. 3c). Thus, the data indicate that DDB2 plays a role on DNA re-replication.

### DDB2 mediated degradation of CDT2 have different effect on CDT1, p21 and SET8

**Fig. 4:**
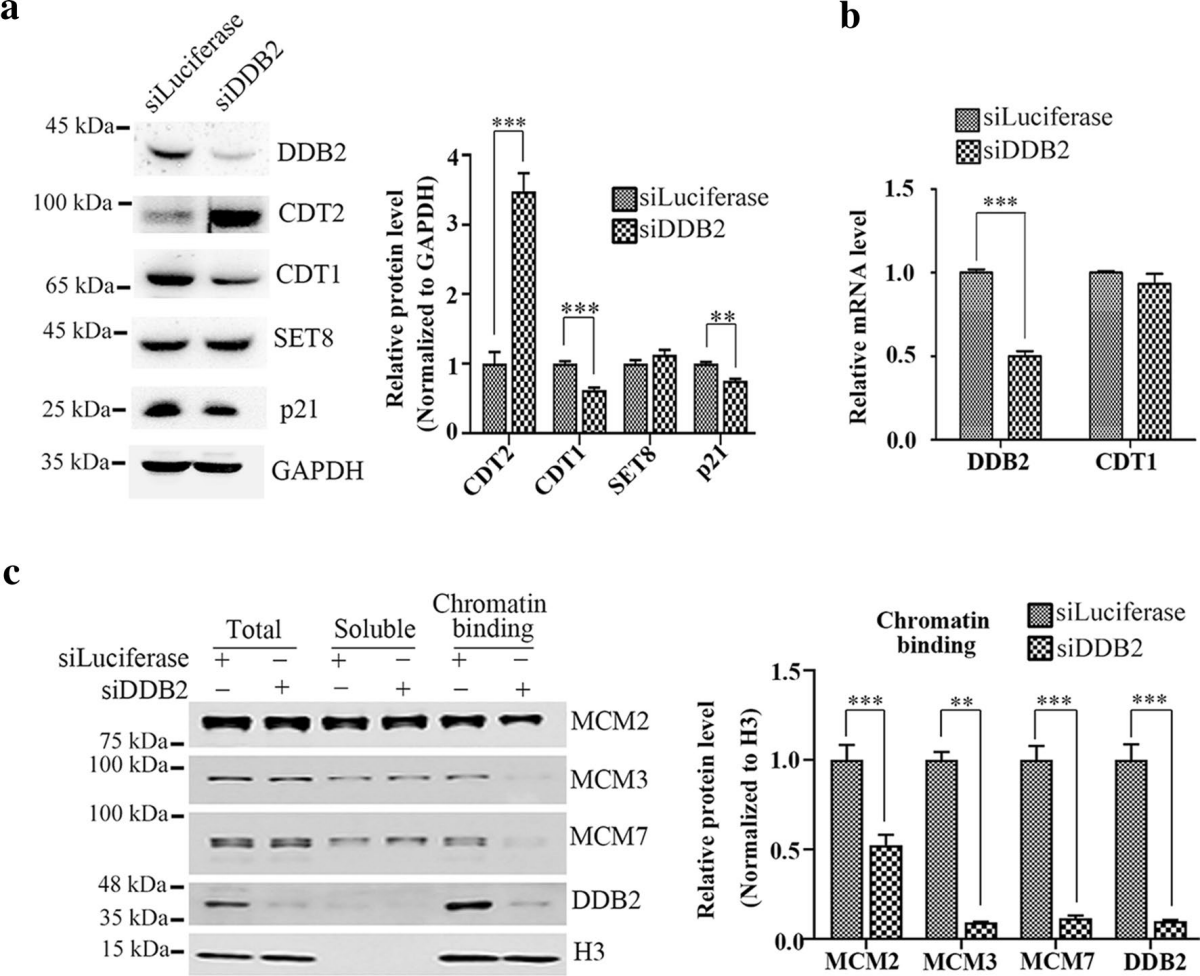
Silence of DDB2 defects the assembly of pre-replication complex. **a** Down regulation of DDB2 reduced the protein level of CDT1 but not SET8. HCT116 cells were treated with indicated siRNAs for 48 h, and then the cells were harvested for Western blot. The relative protein level of p21, CDT1 and SET8 were normalized and plotted on the right panel. *** Denoted P < 0.001, and the error bars indicated SD. **b** The mRNA level of *CDT1* was insensitive to DDB2 depletion. Total RNA was extracted from cells in **a**, and the mRNA levels of *CDT1* and *DDB2* were measured by RT-qPCR. **c** The chromatin recruitment of MCMs was impaired once DDB2 was abolished. HCT116 cells were treated with luciferase and DDB2 siRNAs for 48 h, total cell lysate and cell fractions were blotted with indicated antibodies. The chromatin loading of MCMs was quantified and normalized to histone H3, and plotted on the right panel. ** Denoted P < 0.01, *** denoted P < 0.001

Since depletion of DDB2 can rescue DNA re-replication induced by CDT2 knockdown, we examined the changes of protein levels of CDT1, p21, and SET8—three canonical substrates of CRL4^CDT2^ ubiquitin ligase [5, 8, 43]—after DDB2 silencing, to figure out the mechanism that DDB2 affects DNA re-replication. We found that deletion of DDB2 significantly reduced the protein level of CDT1, and p21was slightly decreased while SET8 was not affected (Fig. 4a), which indicated that degradation of CDT2 mediated by DDB2 has main effect on CDT1 but not p21 and SET8. Since DDB2 can act as a transcriptional regulator, we analyzed mRNA level of CDT1 to clarify whether the down-regulation of CDT1 was transcriptional regulated by DDB2. As shown in Fig. 4b, the mRNA level of CDT1 did not changed after DDB2 silencing (Fig. 4b). It indicated that the decreasing of CDT1 after DDB2 knockdown is due to CDT2 accumulation induced by DDB2 silencing. Considering that CDT1 is the key regulator of pre-replication complex (pre-RC) assembly, our results suggested that CDT2 degradation via CRL4^DDB2^ E3 ubiquitin ligase is in coordination with CDT1 function to license pre-RC assembly.

### DDB2 regulates DNA replication through the assembly of pre-RC

**Fig. 5:**
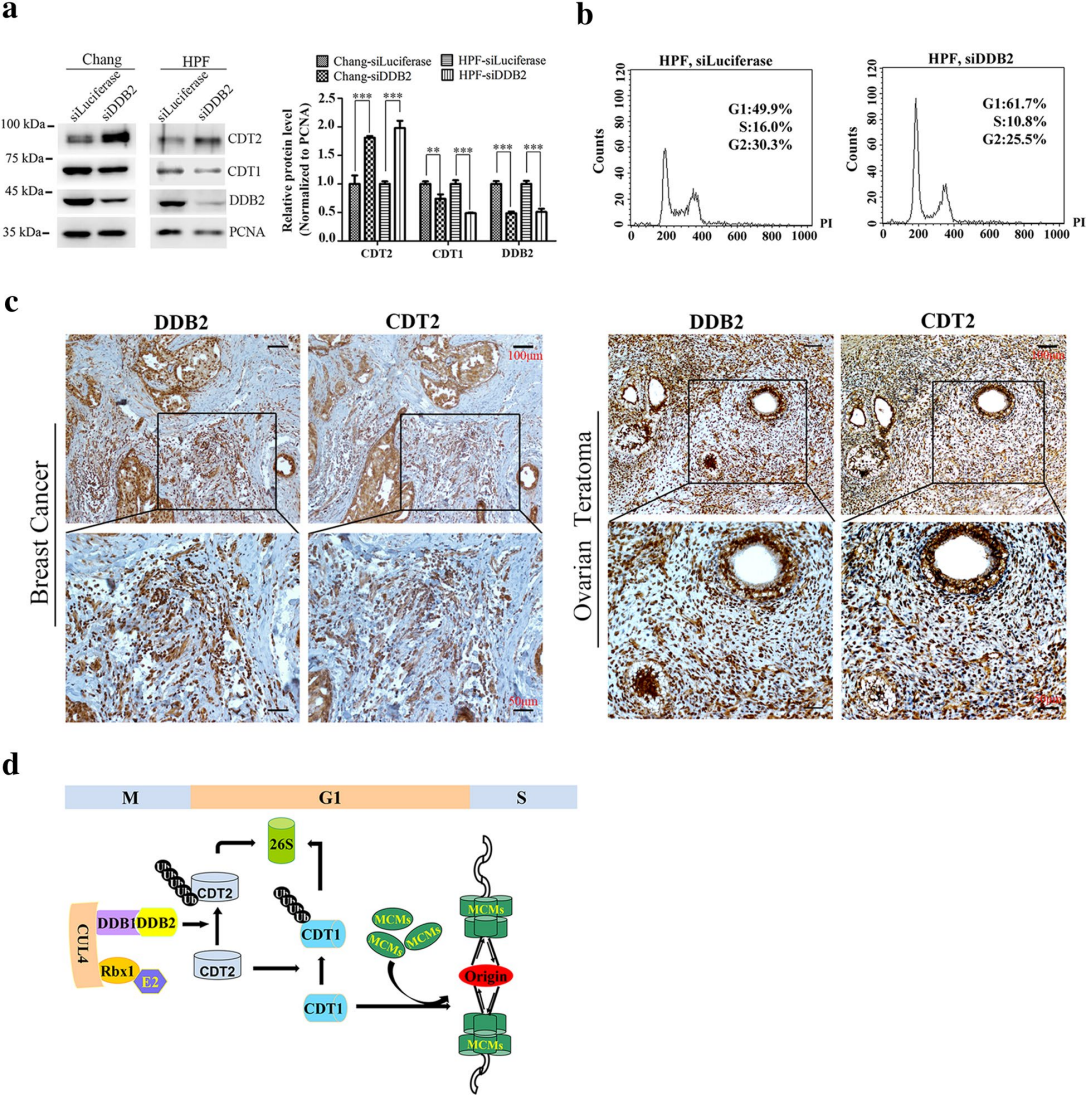
DDB2 mediated proteolysis of CDT2 may corelated with prognostic in cancer patients. **a** DDB2 promoted CDT2 proteolysis in Chang liver and CCC-HPF-1 cells. Chang liver and CCC-HPF-1 cells were treated with siRNAs of luciferase and DDB2 for 48 h, and harvested for Western blot. PCNA was taken as loading control. The relative protein levels of CDT2, CDT1 and DDB2 were normalized and plotted on the right panel. ** Denoted P < 0.01; *** denoted P < 0.001, and the error bars indicated SD. **b** CCC-HPF-1 cells were arrested in G1 phase after DDB2 silencing. FACS analysis of DNA contents in CCC-HPF-1 cells after treatment with siRNAs of luciferase and DDB2 for 48 h. **c** Expression of CDT2 and DDB2 in ovarian teratoma and breast cancer tissues detected by IHC. Scale bar: 50 µm and 100 µm. **d** The schematic model of CRL4^DDB2^ ubiquitin ligase regulates DNA replication initiation through degrading CDT2. In late M and G1 phase, the degradation of CDT2 mediated by CRL4^DDB2^ ubiquitin ligase stabilizes CDT1. Accumulation of CDT1 promotes recruitment of MCMs onto origins and assembly of pre-replication complex

Since knockdown of DDB2 down-regulates the protein level of CDT1, and CDT1 plays a key role on DNA replication initiation by promoting the recruitment of MCM2-7 on chromatin and the assembly of pre-RC, we suppose that the recruitment of MCM2-7 on chromatin will be defected. As expected, the protein levels of MCM2, MCM3 and MCM7 on chromatin were significantly decreased in DDB2 siRNAs treated cells compared with control (luciferase siRNAs treated cells) (Fig. 4c), whereas the total amount of MCMs was not affected (Fig. 4c). Thus, our finding provided a novel mechanism for DNA replication regulation that the degradation of CDT2 directed by CRL4^DDB2^ ubiquitin ligase is required for stabilizing CDT1 to facilitate MCMs recruitment on chromatin during pre-RC assembly (Fig. 5d).

### DDB2 and CDT2 are opposite prognostic markers in cancer patients

To figure out whether it is a common mechanism that CRL4^DDB2^ regulated CDT2 degradation in other cells, we silenced DDB2 in Chang liver cancer cells and human primary lung fibroblast CCC-HPF-1 cells. The similar results were obtained that CDT2 was accumulated after DDB2 knockdown, and subsequent decrease of CDT1 (Fig. 5a). The FACS analysis showed that G1 phase arrest was induced when DDB2 was knockdown in CCC-HPF-1 cells (Fig. 5b), which indicated that DNA replication was delayed. Thus, our data confirmed that it is the common mechanism in normal and cancer cells that CRL4^DDB2^ regulates DNA replication acting as the novel E3 ubiquitin ligase of CDT2. According to the human protein atlas database, DDB2 is a favorable prognostic marker in endometrial cancer, cervical cancer and breast cancer (https://www.proteinatlas.org/ENSG00000134574-DDB2/pathology), whereas CDT2 is the unfavorable prognostic marker in renal cancer and liver cancer (https://www.proteinatlas.org/ENSG00000143476-DTL/pathology), which is fit well with our data.

To further investigate whether the role of CDT2 and DDB2 in cancer patients were related to DDB2 mediated CDT2 degradation, we analyzed expression of CDT2 and DDB2 in ovarian teratoma and breast cancer tissues using immunohistochemistry. As shown in Fig. 5c, in the area where CDT2 was highly expressed, the expression of DDB2 was much lower, while in the area where DDB2 was highly expressed, the expression of CDT2 was very low (Fig. 5c). It matches with our finding that CRL4^DDB2^ is the E3 ubiquitin ligase of CDT2. Thus, our finding provides a new perspective on cancer therapy.

## Discussion and conclusion

It is reported that CDT2 is over-expressed in kinds of cancers, and high expression of CDT2 is related to poor prognosis in cancer patients. Whereas DDB2 is a favorable prognostic marker in cancer patients. Both CDT2 and DDB2 are substrate receptors for CRL4 ubiquitin E3 ligase, but they have opposite functions in tumorigenesis, and the underlining mechanism is still obscure. In this study, we found that DDB2 involves in regulating DNA replication by promoting CDT2 proteolysis to stabilize CDT1, and facilitate the assembly of pre-RC.

In eukaryotic cells, the assembly of pre-RC occurs in late M phase and early G1 phase, and involves the stepwise recruitment of ORCs, CDC6, CDT1 and MCMs on chromatin. The key regulation mechanism to avoid DNA re-replication is the degradation of CDT1 once DNA replication initiated. CRL4^CDT2^ is the key E3 ubiquitin ligases of CDT1. Stabilization of CDT1 by silencing of CDT2 will induce the occurrence of DNA re-replication. DNA re-replication will lead to genome instability.

We found that CRL4^DDB2^ is the novel E3 ubiquitin ligases of CDT2. CDT2 and DDB2 existed in the same immunocomplex. CDT2 was notably accumulated when DDB2 was knocked down and had enhanced ubiquitination when DDB2 was over-expressed (Fig. 1). Although both DDB2 and CDT2 contain PIP box,PCNA is dispensable for the degradation of CDT2 mediated by DDB2 (Fig. 2). Surprisingly, silencing of DDB2 could significantly rescue DNA re-replication induced by CDT2 deletion, which indicated that DDB2 involves in regulating DNA replication (Fig. 3). We found that the stabilization of CDT2 induced by DDB2 knockdown promoted CDT1 degradation and impaired the recruitment of MCMs on the chromatin (Fig. 4). Thus, we proposed that DDB2 regulates DNA replication by degrading CDT2 to stabilize CDT1 and facilitate the recruitment of MCMs, as well as the assembly of pre-RC, and ensure DNA replication initiation in S phase (Fig. 5d).

Cancer cells usually have more DNA contents and encounter more replicative stress and DNA damage, which may induce the apoptotic death of cancer cells, thus cancer cells become addicted to CDT2 because of their enhanced cellular stress. High level of DDB2 can promote CDT2 degradation, which would enhance cellular replicative stress and subsequent apoptotic death of cancer cells. Taken together with that in ovarian and breast cancer tissues, high-expression of CDT2 was along with low level of DDB2 (Fig. 5c), our novel finding may interpret why DDB2-high and CDT2-low patients have better prognosis. Thus, we provide a new perspective for ubiquitin-dependent proteolysis in tumorigenesis, and a novel mechanism for DNA replication regulation.

## Methods

### Cell culture

CCC-HPF-1 cells (3111C0001CCC000107) and Chang liver cells (3131C0001000200009) were obtained from National Infrastructure of Cell Line Resource. CCC-HPF-1 cells were maintained in DMEM with 20% FBS and Chang liver cells were cultured in RPMI 1640 with 10% FBS. HCT116 cells were the gift from Dr. Hui Zhang (Department of Chemistry and Biochemistry, University of Nevada, Las Vegas, Nevada 89154, USA), and cultured in DMEM supplemented with 10% FBS. Cells were authenticated by Shanghai Biowing Biotechnology Co. Ltd, and Mycoplasma were tested after cells were cultured for one weeks using GMyc-PCR Mycoplasma Test Kit (Yeasen Biotech Co. Ltd, Shanghai, China). Cells used for experiments were maintained for one month. Immunostaining was performed as previously described [44].

### Antibodies

Anti-CDT1 (A300-786A), CDT2 (A300-947A), MCM2 (A300-191A) and MCM3 (A300-192A) antibodies were purchased from Bethyl Laboratories Inc. Anti-Histone H3 antibody (ab1791) was from Abcam, and anti-Flag antibody (F1804-200UG) was from Sigma-aldrich. Anti-GAPDH (60004-1-Ig), HA (66006-2-Ig), p21 (10355-1-AP) and SET8 (14063-1-AP) antibodies were purchased from Proteintech. Anti-PCNA (sc-56) and MCM7 (sc-9966) antibodies were purchased from Santa Cruz Biotechnologies. Anti-DDB2 (PA5-79143 and PA5-63568) antibodies were from Invitrogen. Homemade polyclonal antibodies of CDT2 and CUL 1 were gifts from Dr. Hui Zhang (Department of Chemistry and Biochemistry, University of Nevada, Las Vegas, Nevada 89154, USA).

### Chromatin binding proteins isolation

HCT116 cells were trypsinized, washed with PBS and lysed in buffer A (10 mM Hepes pH 7.9, 10 mM KCl, 1.5 mM MgCl_2_, 0.34 M sucrose, 10% glycerol, 0.1% Triton X-100 and protein inhibitors) on ice for 15 min, and centrifuged for 5 min at 2000×*g*. The supernatant was collected as soluble fraction. The precipitation was re-suspended in buffer B (3 mM EDTA, 0.2 mM EGTA, and protein inhibitors). After centrifuged for 5 min at 2000×*g*, the precipitation was digested by micrococcal nuclease in digestion buffer (0.32 M sucrose, 50 mM Tris-Cl pH7.5, 4 mM MgCl2 and 0.1 mM PMSF) for 10 min at 37 °C. After centrifuged for 10 min at 8000×*g*, the supernatant was collected as the chromatin binding proteins fraction.

### Total RNA isolation and real‐time PCR

Cells were harvested with RNAiso Plus (#9109, Takara Biotechnology Co. Ltd., Dalian) and total RNA was isolated according to manufacture. Reverse transcription was performed using Reverse Transcriptase M-MLV (RNase H-) (#2641A, Takara Biotechnology Co. Ltd., Dalian). The relative mRNA levels of target genes were quantified by SYBR Fast qPCR Mix (#RR430S, Takara Biotechnology Co. Ltd., Dalian) in a CFX Connect Real-Time PCR Detection System (Bio-Rad Laboratories, Inc.). GAPDH was taken for normalization. The primers used for real-time PCR were as following: DDB2 forward: GCTGAACATGGACGGCAAAG and DDB2 reverse: CCATCGGGACTGAAACAAGC; CDT2-1 forward: CGTCTCCTATCAGTCCGTAT and CDT2-1 reverse: GGATTCTCAGCCTTCCGTTT; CDT2-2 forward: CGTCTCCTATCAGTCCGTAT and CDT2-2 reverse: TGTCTTTCCGCTCTGTCTCC; CDT1 forward: GTGCTGCGGAGCGTCTTTGT and CDT1 reverse: TGCAGTGATGTGGGCGAGGT; GAPDH forward: ACCACAGTCCATGCCATCA and GAPDH reverse: CAGGGATGATGTTCTGGAGA.

### Co‐immunoprecipitation (Co-IP) and in vivo ubiquitination

For protein co-IP, asynchronized HCT116 cells were suspended in IP buffer (0.5% Nonidet P40, 50 mM Hepes pH 7.5, 150 mM NaCl, 1 mM EDTA, and proteinase inhibitors) on ice for 15 min and centrifuged at 15,500×*g* for 15 min. The supernatant was incubated with indicated primary antibodies overnight at 4 °C. After incubated with protein G beads for 2 h at 4 °C, the immunocomplexes were recovered, and suspended in SDS-sample buffer.

For in vivo ubiquitination, HCT116 cells were co-transfected with 2 µg plasmids expressing HA-tagged ubiquitin and Flag-DDB2 or vectors using polyetherimide. After transfected for 48 h, cells were treated with 10 µg/mL MG132 for 5 h and harvested for co-immunoprecipitation (0.5 % Nonidet P40, 50 mM Hepes pH 7.5, 150 mM NaCl, 0.05 % SDS, 1 mM EDTA, and proteinase inhibitors) using anti-CDT2 antibody. Western blot was performed using anti-HA and Anti-CDT2 antibodies. Anti-HA antibody was used to detect the ubiquitinated CDT2, and anti-CDT2 antibody was used to measure the efficiency of immunoprecipitation.

### Transfection and siRNAs

For transfection, HCT 116 cells were seeded in 6 well plate for 20 h and transfected with indicated plasmids using polyetherimide. Forty-eight hours after transfection, cells were lysed with SDS-sample buffer and proteins were analyzed by Western blot.

Small interfering RNAs were designed and synthesized by GenePharma Company. The siRNA sequences were as following: luciferase: CGTACGCGGAATACTTCGA; CDT2: ACTCCTACGTTCTCTATTA; PCNA: GAUCGAGGAUGAAGAAGGA; DDB2-1: CTCCAGAGTTGGTGACACA; DDB2-2: GATGGAAACTCAGGGAAGA; DDB2-3: GAGCGAGAUCCGAGUUUAC.

For siRNA mediated silencing, HCT116 cells were transfected with 50 nM siRNAs for 48 h using DharmaFECT Transfection Reagent (#T-2001-03, Thermo Fisher Scientific Inc.) according to manufacturer’s instructions, and cell lysate was analyzed by Western blot.

### Cell synchronization and flow cytometry analysis

Cell synchronization was performed as described. G1/S arrest was achieved by double thymidine (2.5 mM) treatment (16 h treament–12 h release–16 h treatment). S phase was obtained from G1/S release for 4 h. Small interfering RNA mediated silencing was performed 8 h before thymidine was added.

For flow cytometry analysis, cells were fixed with ice-cold 70 % ethanol for 2 h at 4 °C. After rinsed with PBS, cells were incubated in staining buffer (25 µg/mL propidium iodide, 1% Trion X-100 and 50 µg/mL RNAase) for 30 min at 37 °C, and analyzed by FACS (Cytomis FC 500, Beckman Coulter). DNA contents were evaluated with FlowJo7.6.5.

### Immunohistochemistry

Human ovarian teratoma tissue sections and breast cancer tissue sections were prepared by Shenzhen Second People’s Hospital, and proved by the Ethics Committee of Shenzhen Second People’s Hospital in accordance with the principles of the 1964 Helsinki declaration. Immunohistochemistry (IHC) was performed using horseradish peroxidase/3,3′-diaminobenzidine (DAB) (ABC) detection IHC Kit (ab64261, abcam). Briefly, The slides were heated at 60 °C for 90 min, deparaffinized in xylene, rehydrated with 100%, 90%, 80% and 70% ethanol, and immersed in methanol with 3% H_2_O_2_ (hydrogen peroxide) for 10 min at room temperature to inactivate endogenous peroxidase. Antigens were heat-retrieved in sodium citrate buffer (10 mM sodium citrate and 0.05% Tween 20, pH 6.0) at 100 °C for 8 min. After blocked with 10 % serum for 30 min at 25 °C, the slides were incubated with primary antibodies against CDT2 (dilution 1:150) and DDB2 (dilution 1:150) overnight at 4 °C. Incubated with biotin-conjugated secondary antibody for 20 min at 25 °C, washed with PBS, and then incubated with streptavidin peroxidase for 15 min at room temperature, the sections were stained with DAB and counterstained in hematoxylin. After dehydration and coverslip, images were captured under microscope (Olympus IX73) using cellSens Dimension program.

### Quantification and statistical analysis

To quantify and compare the protein band densities in the Western blots shown in the figures, the blots was quantified by Gel-Pro Analyzer (version4.0, Media Cybernetics, Inc.), which is the software for area density analyzation. The band density of each protein in various samples was normalized to loading controls.

All experiments were performed at least three times. Data were presented as mean ± SD. The mean was generated from triplicate experiments and SD was the standard deviation. Paired two-side Student’s t-test was performed to measure the significance of the difference between control group and experimental group, and p < 0.05 was considered as significant. * Denotes p < 0.05, ** denotes p < 0.01, and *** denotes p < 0.001. GraphPad Prism 5.0 was used to generate the plots.

## Data Availability

All data and materials are available. Not applicable.
